# Cerebral tissue oxygen saturation and its potential relationship with neurodevelopmental delay in pediatric liver transplant recipients

**DOI:** 10.3389/fped.2024.1416020

**Published:** 2025-01-07

**Authors:** Yichen Fan, Qianling Pan, Henghua Su, Zhongchan Pu, Linjie Zhu, Bo Qi, Diansan Su, Liqun Yang, Dan Huang, Weifeng Yu

**Affiliations:** ^1^ Department of Anesthesiology, Renji Hospital, School of Medicine, Shanghai Jiaotong University, Shanghai, China; ^2^ Department of Operation Room, Renji Hospital, School of Medicine, Shanghai Jiaotong University, Shanghai, China

**Keywords:** cerebral tissue oxygen saturation, postoperative neurodevelopmental delay, pediatric living-donor liver transplantation, Ages Stages Questionnaires, perioperative

## Abstract

**Objective:**

To discover the potential association between diminished intraoperative average SctO_2_ levels and postoperative neurodevelopmental delays among patients after pediatric living-donor liver transplantation.

**Study design:**

Patients undergoing living-donor liver transplantation were recruited for this trial. The neurodevelopment status of patients was assessed using the Ages Stages Questionnaires. The primary outcome was the occurrence of neurodevelopmental delay among patients at different intervals following pediatric liver transplantation. Secondary outcomes included the duration of mechanical ventilation, rates of re-intubation, length of ICU stay, postoperative hospitalization, and intraoperative comparisons of mean arterial pressure (MAP), arterial partial pressure of oxygen (PaO_2_), arterial partial pressure of carbon dioxide (PaCO_2_), and hemoglobin (Hb) concentration.

**Results:**

A total of 119 patients were included in the statistical analysis and assigned to high saturation group (HS) and low saturation group (LS) according to the average intraoperative cerebral tissue oxygen saturation values. Following adjustment for PELD scores, significant differences between the two groups were observed for the incidence of neurodevelopmental delay in communication at 1 and 3 months follow-up (*P* = 0.019 and *P* = 0.020, respectively), fine motor at six months follow-up (*P* = 0.014), and problem-solving abilities at one year follow-up (*P* = 0.047). Moverover, the length of ICU stay (*P* = 0.009) and postoperative hospitalization (*P* = 0.029) in LS group were also significant prolonged.

**Conclusion:**

This prospective observational study revealed that the patients with low average SctO_2_ values were more predisposed to experiencing postoperative neurodevelopment delays, suggesting a potential association between decreased average SctO_2_ and neurodevelopmental delay.

## Introduction

Biliary atresia represents a rare infancy ailment, with a global pediatric incidence estimated at approximately 1 per 10,000 births (1, 2). Treatment options for pediatric biliary atresia typically encompass the Kasai procedure, liver transplantation, and adjunctive pharmacotherapies. Among these treatments, the most effective therapy is pediatric liver transplantation. Recent advancements in surgical techniques have significantly improved the overall survival rates post-surgery. The 1-year survival rate among patients of living-donor liver transplantation could exceed 95%, marking a considerable improvement over historical benchmarks. Nevertheless, despite these advancements, about 20% of patients still experience unfavorable prognoses and contend with serious postoperative complications (3).

Neurological impairment emerged as one of the foremost postoperative complications. Previous study found that about 46% of patients may have experienced neurological damage, potentially progressing to cognitive dysfunction and long-term neurodevelopmental delays following pediatric liver transplantation (4). Cerebral oxygen metabolism imbalance has been identified as a contributing factor to such neurological complications (5). Monitoring SctO_2_ utilizing near-infrared spectroscopy (NIRS) facilitated real-time assessment of oxygen saturation levels within localized brain regions, offering insights into cerebral oxygen metabolism dynamics (6). Previous study (7) had linked decreased SctO_2_ levels with neuro-dysplasia in children undergoing cardiac surgery. However, the precise relationship between intraoperative SctO_2_ levels and long-term neurodevelopmental outcomes subsequent to pediatric liver transplantation remained unclear.

In our study, we hypothesize that diminished intraoperative average SctO_2_ levels might have associated with postoperative neurodevelopmental delays among patients undergoing pediatric living-donor liver transplantation.

## Materials and methods

### Study design

This prospective observational study adhered to the principles outlined in the Declaration of Helsinki and obtained approval from the ethics committee of Renji Hospital (SK2020-059). It was registered at Clinical Trail.gov (NCT04518332). All patients enrolled in this study signed the informed consent form prior to their participation.

### Study population

Eligible participants were patients aged 3 months to 3 years diagnosed with congenital biliary atresia and scheduled for living-donor liver transplantation. Exclusion criteria encompassed: (1) combined organ transplantation; (2) cutaneous infection or head trauma; and (3) concurrent participation in other clinical trials.

### Perioperative management

The standardized anesthesia protocol was performed in all patients. Prior to anesthesia induction, patients adhered to fasting guidelines (8). Pre-anesthesia assessments included peripheral oxygen saturation (SpO_2_) and electrocardiography monitoring. Anesthesia induction entailed inhalation of 8 vol.% sevoflurane with 100% oxygen at 8 L/min. Following loss of consciousness, intravenous induction was initiated via peripheral intravenous route, primarily comprising midazolam 0.1 mg/kg, sufentanil 0.5 µg/kg, and rocuronium 1 mg/kg. Tracheal intubation, facilitated by video laryngoscopy, allowed for intermittent positive-pressure ventilation. The oxygen concentration was set at 60%, and the ventilator mode was adjusted to pressure-controlled ventilation. Airway pressure was regulated within the range of 12–25 cmH_2_O (1 cmH_2_O = 0.098 kPa) to maintain the end-expiratory carbon dioxide partial pressure at 35–45 mmHg, while the respiratory rate was set between 16 and 20 breaths per minute. Continuous monitoring of arterial blood pressure and central venous pressure was achieved through punctures of the arterial line and right internal jugular vein, respectively. Body temperature was maintained above 36°C using insulation blankets and a fluid warmer (Smith Medical, MN, USA).

Intraoperative monitoring included cerebral tissue oxygen saturation, with NIRS sensors placed on the forehead of patients after proper cleaning. Simultaneous NIRS data were collected and analyzed using the specific brain/body oximeter system (INVOS 5100C, MN, USA) throughout the procedure.

Sevoflurane, rocuronium, and sufentanil were used for anesthesia maintenance. Routine analysis of blood gas was performed at specific time points: at the beginning of surgery, during the anhepatic phase (30 min after clamping), and 10 min and 2 h after re-perfusion. Electrolyte, glucose level, and acid-base balance were adjusted to normal level based on blood gas results. Only crystalloids, 5% glucose, and 20% albumin were used. Red blood cells transfusion was administered only if hemoglobin levels fell below 7 g/dl. Vasoconstrictors, including epinephrine and norepinephrine, were used to maintain blood pressure. Following surgery, patients were transferred to the ICU for further treatment.

### Assessment of neurodevelopment and follow-up

The neurodevelopment status of patients was assessed using the Ages Stages Questionnaires (ASQ-3) scale by the same trained evaluator at five time points: baseline (one day before surgery), and at one, three, six, and twelve months after surgery, respectively. The ASQ scale, designed by Professors Squires and Bricker of the University of Oregon, comprised five sub-scales including communication, gross motor, fine motor, problem-solving, and social skills, completed by parents (9). As mentioned in the ASQ User' s Guide (10), the cutoff point was defined as 2 standard deviation below the mean of normal sample. The scores for each sub-scale were compared with cutoff point to identify the possible neurodevelopmental delays.

### Outcomes and variables

The primary outcome assessed in this study was the incidence of neurodevelopmental delay among patients at different intervals following pediatric liver transplantation. Secondary outcomes included the duration of mechanical ventilation, the occurrence of re-intubation, length of ICU stay, postoperative hospitalization duration, and comparisons of intraoperative mean arterial pressure (MAP), arterial partial pressure of oxygen (PaO_2_), arterial partial pressure of carbon dioxide (PaCO_2_) and hemoglobin (Hb) concentration. Other perioperative variables included sex, age, body mass index (BMI), pediatric end-stage liver disease score (PELD), average SctO_2_, surgical duration, anhepatic phase duration, cold ischemia time, intraoperative blood transfusion, and laboratory test results including albumin, alanine transaminase (ALT), total bilirubin (TBIL), Hb, international normalized ratio (INR), and creatinine.

### Statistical analyses

Statistical analyses were conducted using R software (version 4.0.2). The distribution of data was assessed initially using the Shapiro-Wilk test., with a *P* > 0.05 indicating conformity to a normal distribution. Categorical variables were reported as numbers (percentages), while continuous variables were expressed as the mean (standard deviation) or median [interquartile range] depending on the normality of the data. Student *t* test and Mann–Whitney *U* test were used to analyze continuous variables, whereas the chi-squared test was used to compare categorical variables. A value of *P* < 0.05 was considered as statistical significant.

## Results

### Participants

**Figure 1:**
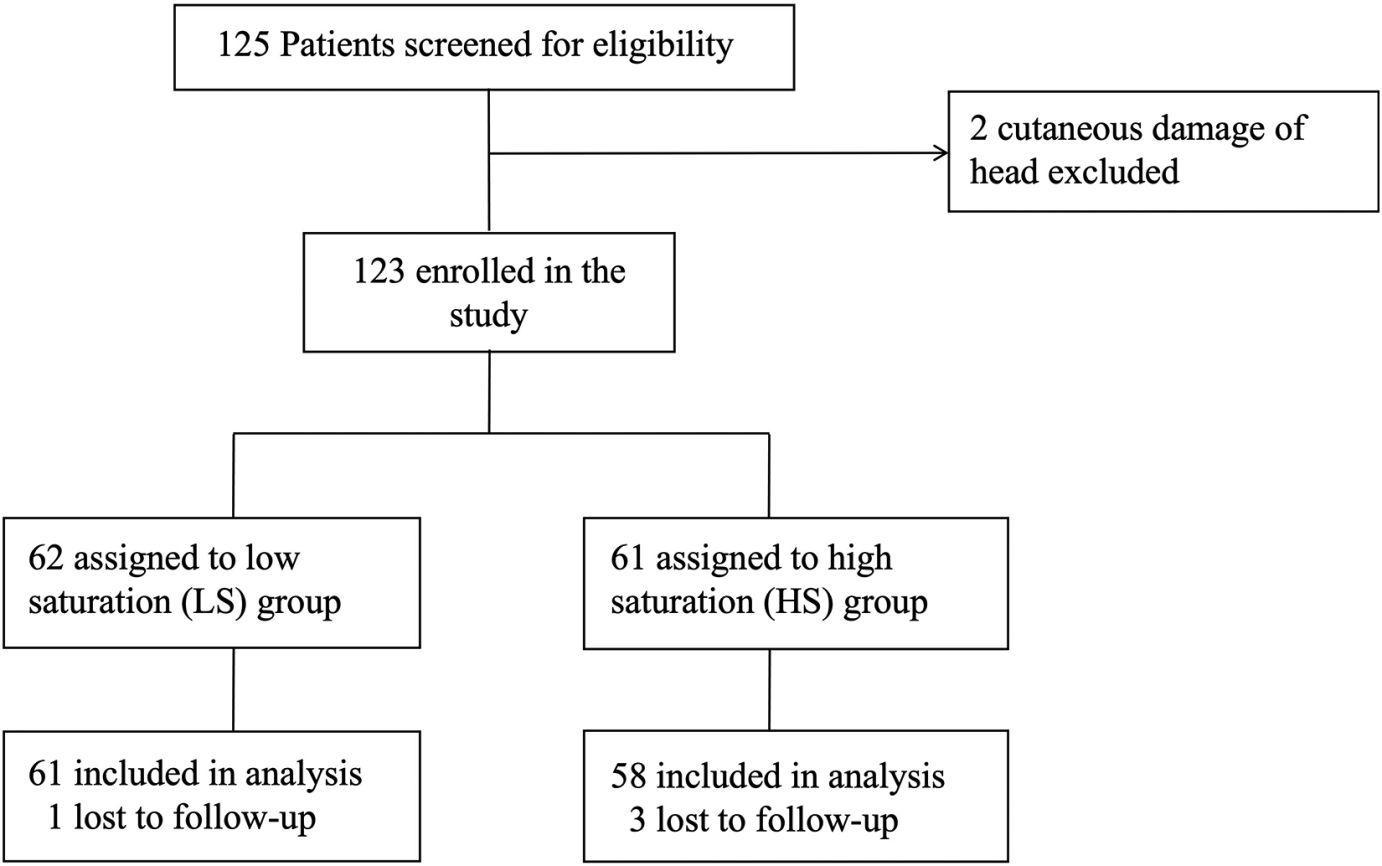
Flowchart of the study.

Between September 2020 to September 2021, 125 patients undergoing pediatric living-donor liver transplantation were screened for eligibility, This sample size is relative small but conforming to the requirements of neurodevelopmental measurement (11). A total of 123 patients consented and were recruited into the study after excluding 2 patients due to head cutaneous damage. Following assignment was made after the surgery, based on intraoperative average SctO_2_. 62 patients were assigned to the low saturation (LS) group (SctO_2_ < 50%), while 61 patients were assigned to the high saturation (HS) group (SctO_2_ ≥ 50%). 4 patients (1 in LS group and 3 in HS group) were dropped out due to loss of follow-up. One year follow-up for the last patient ended in July 2022. Finally, 119 patients were included in the statistical analysis (Figure 1).

### Characteristics and preoperative variables

The characteristics and peri-operative variables of patients, including preoperative ASQ assessment results, are shown in Table 1. Most peri-operative variables were well balanced between the two groups. However, significant differences were observed in age [5.0 [5.0, 7.0] vs. 7.5 [6.0, 12.3]; *p* < 0.001], PELD score [16.6 [11.5, 21.6] vs. 10.1 [−2.0, 15.8]; *p* < 0.001], INR [1.4 [1.1, 1.8] vs. 1.1 [1.0, 1.4]; *p* = 0.001], TBIL [240.1 [179.0, 346.2] vs. 109.2 [23.9, 238.1]; *p* < 0.001], and RBC transfusion [200.0 [200.0–250.0] vs. 200.0 [100.0–200.0]; *p* = 0.002].

**Table 1:** Comparisons of patient characteristics and perioperative variables between the two groups.

Variables	Group		*P* value
	LS (*n* = 61)	HS (*n* = 58)	
Male (%)	28 (45.9%)	23 (39.7%)	0.491
Age (month)	5.0 [5.0, 7.0]	7.5 [6.0, 12.3]	<0.001
BMI (kg/m^2^)	16.1 (2.0)	16.2 (2.0)	0.901
PELD score	16.6 [11.5, 21.6]	10.1 [−2.0, 15.8]	<0.001
Albumin (g/L)	35.5 (4.5)	36.3 (4.9)	0.340
ALT (U/L)	141.0 [97.0, 224.5]	140.0 [76.0, 187.0]	0.277
TBIL (µmol/L)	240.1 [179.0, 346.2]	109.2 [23.9, 238.1]	<0.001
Creatinine	13.0 [12.0, 16.0]	15.0 [13.0, 16.3]	0.202
INR	1.4 [1.1, 1.8]	1.1 [1.0, 1.4]	0.001
Hb (g/L)	94.4 (15.9)	99.2 (15.2)	0.088
PLT (10^9^/L)	232.0 [165.5, 337.0]	212.5 [169.5, 265.5]	0.271
Duration of surgery (min)	332.5 (45.1)	349.4 (54.8)	0.063
Anhepatic phase (min)	34.0 [27.0, 38.0]	34.5 [30.8, 38.3]	0.283
Cold ischemia time (min)	60.0 [51.0, 73.0]	63.5 [53.8, 79.5]	0.187
RBC transfusion (ml)	200.0 [200.0–250.0]	200.0 [100.0–200.0]	0.002
ASQ-3 assessment			
Communication (%)	31 (50.8%)	28 (48.3%)	0.782
Gross motor (%)	33 (54.1%)	37 (63.8%)	0.283
Fine motor (%)	20 (32.8%)	16 (27.6%)	0.537
Problem-solving (%)	22 (36.1%)	13 (22.4%)	0.102
Social skill (%)	34 (55.7%)	32 (55.2%)	0.951

The data are presented as the mean (standard deviation), median [interquartile range] and number (percentage).

BMI, body mass index; PELD, pediatric end-stage liver disease; ALT, alanine aminotransferase; TBIL, total bilirubin; INR, international normalized ratio; Hb, hemoglobin; PLT, platelet; RBC, red blood cell; ASQ, ages & stages questionnaires.

### Primary outcome

**Figure 2:**
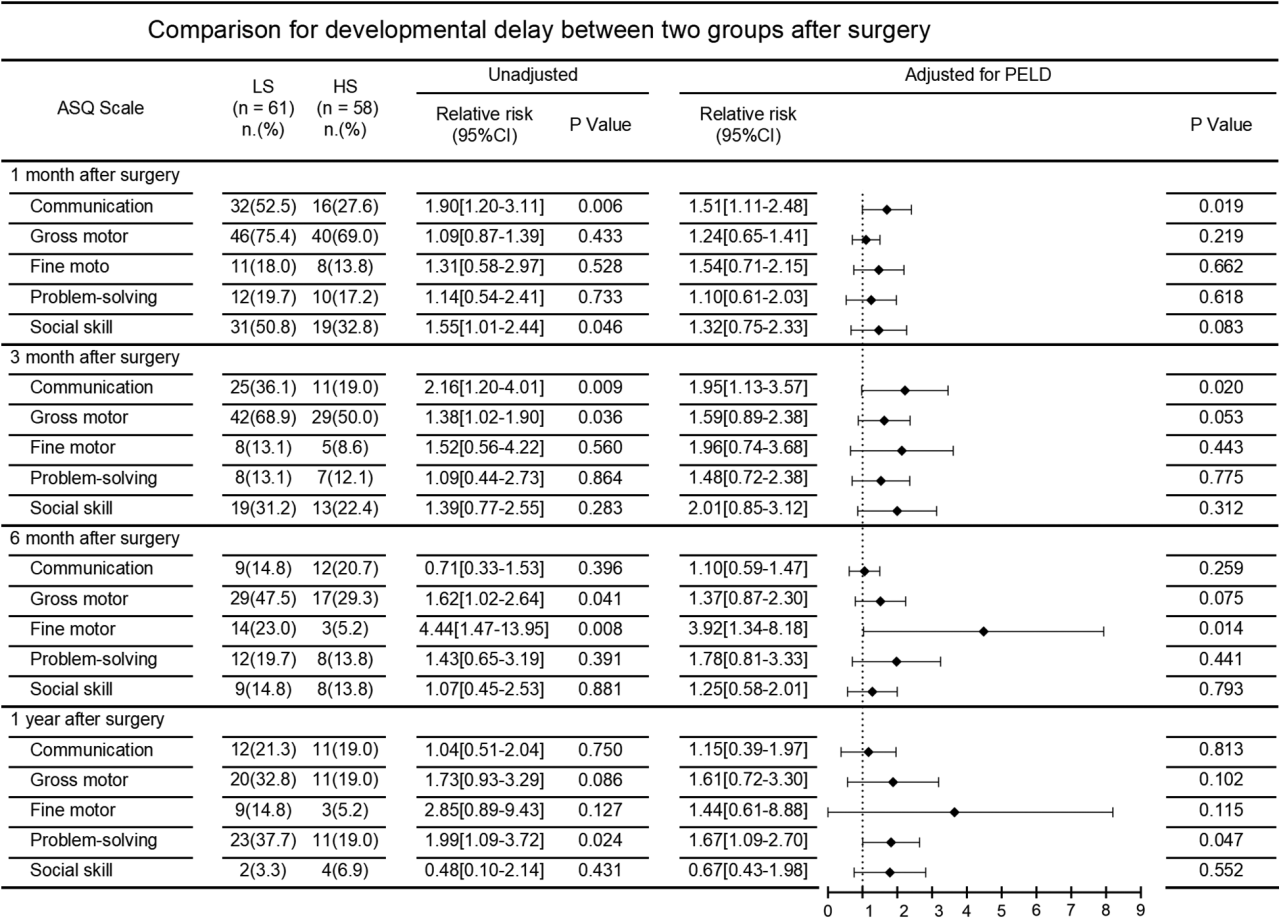
Comparison for developmental delay between two groups after surgery.

The incidence of neurodevelopmental delay after surgery in the two patient groups is demonstrated in Figure 2. At the 1-month postoperative follow-up, communication [52.5% vs. 27.6%; unadjusted RR, 1.90 (95%CI, 1.20–3.11)] and social skill [50.8% vs. 32.8%; unadjusted RR, 1.55 (95%CI, 1.01–2.44)] were significantly better in the HS group compared to the LS group. At the 3-month postoperative follow-up, communication [36.1% vs. 19.0%; unadjusted RR, 2.16 (95%CI, 1.20–4.01)] and gross motor skills [68.9% vs. 50.0%; unadjusted RR, 1.38 (95%CI, 1.02–1.90)] in the LS group were far worse compared to that in the HS group. Similarly, at the 6-month follow-up, gross motor [47.5% vs. 29.3%; unadjusted RR, 1.62 (95%CI, 1.02–2.64)] and fine motor skills [23.0% vs. 5.2%; unadjusted RR, 4.44 (95%CI, 1.47–13.95)] in the LS group were significant worse. At the 1-year follow-up, patients in the LS group showed poorer problem-solving abilities [37.7% vs. 19.0%; unadjusted RR 1.99 (95%CI 1.09–3.72)].

After adjusting for PELD score, comparisons between the LS group and HS group still showed significant differences in the incidence of neurodevelopmental delay in communication at 1 and 3 months follow-up [adjusted RR 1.51 [95%CI 1.11–2.48] and adjusted RR 1.95 [95%CI 1.13–3.57], respectively], in fine motor skills at six months follow-up [adjusted RR 3.92 (95%CI 1.34–8.18)], and in problem-solving at 1 year follow-up [adjusted RR 1.67 (95%CI 1.09–2.70)].

### Secondary outcomes

Comparisons of postoperative variables between the LS group and HS group are shown in Table 2. There were no significant differences in mechanical ventilation time [20.4 h IQR [19.2–38.5] vs. 19.9 h IQR[18.5–42.6]; *P* = 0.343] and the incidence of re-intubation (1.6% vs. 1.7%; *P* = 0.971). However, the length of ICU stay in the LS group was 6 days [IQR(5.0–7.0)] compared to 5 days [IQR(5.0–6.0)] in the HS group, showing significant difference (*P* = 0.009). There was a significant difference in the length of postoperative hospitalization between the two groups [35.0 days IQR [28.5–43.0] vs. 31.0 days IQR[23.0–37.0]; *P* = 0.029].

**Table 2:** Comparisons of postoperative variables between two groups.

Variables	LS (*n* = 61)	HS (*n* = 58)	*P* value
Mechanical ventilation time (h)	20.4 [19.2, 38.5]	19.9 [18.5, 42.6]	0.343
Re-intubation (%)	1 (1.6%)	1 (1.7%)	0.971
Length of ICU stay (day)	6.0 [5.0, 7.0]	5.0 [5.0, 6.0]	0.009
Hospitalization (day)	35.0 [28.5, 43.0]	31.0 [23.0, 37.0]	0.029

The intra-operative vital signs and blood gas results are shown in Table 3. The MAP differed significantly between the LS group and HS groups at the beginning of surgery (*P* = 0.001) and 2 h after reperfusion (*P* = 0.011). The Hb concentration in the LS group was significantly lower than that in the HS group at the beginning of surgery (*P* = 0.015), 10 min after reperfusion (*P* = 0.001), and 2 h after reperfusion (*P* = 0.001). No difference was observed in PaCO_2_ and PaO_2_ at different periods during surgery.

**Table 3:** Comparisons of vital signs and blood gas between two groups.

Variables	Group		*P* value
	LS (*n* = 61)	HS (*n* = 58)	
Beginning of the surgery			
MAP (mmHg)	57.0 [54.0, 62.0]	64.0 [57.0, 68.0]	<0.001
PaCO_2_ (mmHg)	43.1 [36.4, 49.8]	43.7 [38.0, 49.4]	0.602
PaO_2_ (mmHg)	220.0 [182.3, 270.8]	238.0 [202.5, 263.3]	0.524
Hb (g/dl)	7.6 [6.7, 9.1]	8.5 [7.5, 9.9]	0.015
30 min after clamping			
MAP (mmHg)	62.5 [58.0, 69.0]	60.0 [52.5, 68.0]	0.210
PaCO_2_ (mmHg)	35.9 [32.9, 40.5]	36.0 [33.2, 40.1]	0.982
PaO_2_ (mmHg)	301.0 [273.0, 327.0]	295.0 [269.0, 319.0]	0.715
Hb (g/dl)	7.7 [6.6, 8.8]	8.1 [6.5, 9.7]	0.095
10 min after re-perfusion			
MAP (mmHg)	57.0 [51.0, 64.8]	56.0 [49.8, 64.3]	0.601
PaCO_2_ (mmHg)	38.9 [34.0, 43.8]	38.0 [33.1, 42.9]	0.296
PaO_2_ (mmHg)	298.0 [279.0, 325.5]	303.0 [272.5, 320.5]	0.639
Hb (g/dl)	7.3 [5.9, 8.7]	8.2 [6.9, 9.5]	0.001
2 h after re-perfusion			
MAP (mmHg)	52.0 [48.0, 57.5]	57.0 [51.0, 62.3]	0.011
PaCO_2_ (mmHg)	39.7 [37.1, 42.8]	39.1 [36.7, 42.5]	0.744
PaO_2_ (mmHg)	281.0 [257.0, 303.0]	297.0 [267.3, 314.8]	0.141
Hb (g/dl)	7.1 [5.7, 8.5]	7.9 [6.6, 9.2]	0.001

The data are presented as the median [interquartile range].

MAP, mean arterial pressure; PaCO_2_, arterial partial pressure of carbon dioxide; PaO_2_, arterial partial pressure of oxygen; Hb, hemoglobin.

## Discussion

This prospective observational study revealed that patients with low cerebral tissue oxygen saturation (LS group, SctO_2_ < 50%) during pediatric living-donor liver transplantation exhibited delayed neurodevelopment in terms of communication, fine motor, and problem-solving skills within 1 year follow-up, compared to those with high cerebral tissue oxygen saturation (HS group, SctO_2_ ≥ 50%). Moreover, the length of ICU stay and postoperative hospitalization were also significantly prolonged in the LS group.

Neurodevelopment outcomes following pediatric transplantation have long been a concern for clinicians and family members of patients. Previous study (12) found that patients undergoing pediatric liver transplantation would experience deficits in various congnitive dunctions, such as visual-spatial abilities, reasoning abilities, and verbal and operational intelligence. A multicenter observational study (13) also revealed that about 60% of parents perceived a decline in their children's cognition and performance after pediatric liver transplantation. Another study (14) found that out of 46 patients who underwent liver transplantation for biliary atresia, 12 required special education, significantly higher than the societal average of 2.4%. These findings aligned with our results, suggesting that a proportion of patients experience a certain degree of neurodevelopment delay in communication skills, motor skills, ability of problem-solving and social interaction following pediatric liver transplantation.

The mechanisms underlying neurodevelopment delay post-transplantation primarily include intraoperative cerebral hypoperfusion (15) and reperfusion injury (16). These changes would lead to short-term neurological damage, which may progress to neurodevelopmental delay detectable by SctO_2_ monitoring. SctO_2_ monitor has been proved to reduce the incidence of neurological damage and complications (17). Hyttel-Sorensen et al. discovered that SctO_2_ monitoring significantly decreased the incidence of cerebral hypoxia in premature infants (18). Despite this, the optimal SctO_2_ value during surgery remains unclear. According to 2019 edition of Chinese consensus guideline for cerebral protection in the perioperative period of cardiac surgery (19), it was recommended to maintain intraoperative SctO_2_ above 50% and closely monitor values below this threshold. Based on this guideline, the cutoff point of SctO_2_ in our study was set at 50%. In our study, we found that patients with SctO_2_ below 50% were more likely to experience postoperative neurodevelopment delay. However, further research is needed to determine the optimal range of SctO_2_ for pediatric liver transplantation patients.

Several factors can influence the value of SctO_2_, with serum total bilirubin level being a significant contributor. SctO_2_ values are decreased in case of increased serum bilirubin concentration (20, 21). Song et al. found an independent relationship between elevated bilirubin levels and SctO_2_ < 50% (22). In addition, the value of SctO_2_ were also influenced by vital signs and blood gas, especially MAP, PaO_2_, PaCO_2_ and Hb concentrations (23, 24). Ballard et al. (25) revealed that the value of SctO_2_ can be elevated by increasing blood pressure, improving hemoglobin level, adjusting respiratory parameters, and oxygen concentration. Our study observed similar results, with patients in the LS group exhibiting significantly lower MAP and Hb concentrations compared to the HS group. These findings suggested that interventions aimed at optimizing these factors may help prevent neurodevelopmental delay in pediatric liver transplantation patients.

Continuous monitoring of SctO_2_ intraoperatively is essential, particularly in pediatric liver transplantation, which is characterized by its complexity and a higher likelihood of lower SctO_2_ levels compared to other surgical procedures (26). Natalia et al. reported that the values of SctO_2_ during pediatric liver transplantation can exhibit considerable variability, ranging from 20% to 90% throughout the procedure (27). Several factors might lead to decreased SctO_2_ levels during pediatric living-donor liver transplantation. First, the preoperative condition of patients is often compromised, and intraoperative hemorrhage is relatively common in this complicated surgery, leading to hypotension and reduced hemoglobin levels, consequently lowering SctO_2_ levels. Second, during the anahepatic phase of liver transplantation, clamping of the portal vein and inferior vena cava significantly reduced cardiac output, resulting in decreased cerebral blood flow (28) and SctO_2_ (26). Third, cerebral autoregulation in young children was less efficient, especially under sevoflurane anesthesia (29). Pether et al. revealed that cerebral perfusion was pressure-dependent in young pediatric patients during sevoflurane anesthesia, suggesting the limited efficiency of the cerebral blood flow autoregulation (30). This made the nerve system more susceptible to hypovolemia or hypoperfusion.

As mentioned above, this is an observational study. No additional intervention were taken and all patients received standardized anesthesia management during the surgery. Great effort has been made to ensure patient stability, such as blood transfusion, usage of vasoactive agents and changes in ventilation strategies. However, due to the complexity of liver transplantation and different preoperative condition of patients, fluctuations in vital signs during the surgery are still inevitable, the value of SctO_2_ also changed accordingly. Though this study, we considered that more attention should be paid on SctO_2_ monitor to optimize anesthesia management.

### Limitations

This study has several limitations that need to be acknowledged. First, the total sample size was small, which might have introduced statistical bias. Second, the follow-up duration was only 1 year, potentially insufficient to fully capture the long-term neurological developmental status of patients after surgery. Moreover, the neurological developmental status during the year following surgery might be influenced by various factors such as nutritional intake (31) and parental care (32), which were not accounted for in our study. Third, due to the unique preoperative conditions of patients, there were significant differences in baseline characteristics such as age, total bilirubin, INR, and PELD score between the two groups. To address this bias, the result was adjusted by the PELD score, which mainly includes age, total bilirubin, and INR. Fourth, the ASQ scale used to evaluate neurodevelopment status in this study was testified to be consistent with other measurements (33). However, it was still a subjective evaluation scale that relied on parental attention and cognitive abilities, which may introduce some degree of deviation in the results. Despite these limitations, our study provides valuable isights that can inform the design of future prospective multicenter studies aimed at addressing these challenges.

## Conclusions

This prospective observational study found that decreased average SctO2 may be associated with neurodevelopmental delay following pediatric living-donor liver transplantation. The occurrence of neurodevelopmental delay can be partially prevented by maintaining SctO2 over 50%. Monitoring SctO2 levels can help clinicians to detect potential neurological damage such as cerebral hypoxia. We also recommend the utilization of SctO2 monitoring routinely during the perioperative period of pediatric liver transplantation.

## Data Availability

The raw data supporting the conclusions of this article will be made available by the authors, without undue reservation.
